# An Investigation of the Role of Common and Rare Variants in a Large Italian Multiplex Family of Multiple Sclerosis Patients

**DOI:** 10.3390/genes12101607

**Published:** 2021-10-13

**Authors:** Nadia Barizzone, Rachele Cagliani, Chiara Basagni, Ferdinando Clarelli, Laura Mendozzi, Cristina Agliardi, Diego Forni, Martina Tosi, Elisabetta Mascia, Francesco Favero, Davide Corà, Lucia Corrado, Melissa Sorosina, Federica Esposito, Miriam Zuccalà, Domizia Vecchio, Maria Liguori, Cristoforo Comi, Giancarlo Comi, Vittorio Martinelli, Massimo Filippi, Maurizio Leone, Filippo Martinelli-Boneschi, Domenico Caputo, Manuela Sironi, Franca Rosa Guerini, Sandra D’Alfonso

**Affiliations:** 1 Department of Health Sciences, CAAD (Center for Translational Research on Autoimmune and Allergic Diseases), University of Eastern Piedmont, 28100 Novara, Italy; chiara.basagni@uniupo.it (C.B.); martina.tosi@uniupo.it (M.T.); lucia.corrado@med.uniupo.it (L.C.); miriam.zuccala@med.uniupo.it (M.Z.); 2 Bioinformatics, Scientific Institute IRCCS E.MEDEA, 23842 Bosisio Parini, Italy; rachele.cagliani@bp.lnf.it (R.C.); diego.forni@lanostrafamiglia.it (D.F.); manuela.sironi@bp.lnf.it (M.S.); 3 Laboratory of Genetics of Neurological Complex Disorders, Division of Neuroscience, IRCCS San Raffaele Scientific Institute, 20132 Milan, Italy; clarelli.ferdinando@hsr.it (F.C.); mascia.elisabetta@hsr.it (E.M.); sorosina.melissa@hsr.it (M.S.); esposito.federica@hsr.it (F.E.); 4 IRCCS Fondazione Don Carlo Gnocchi ONLUS, 20148 Milan, Italy; lmendozzi@dongnocchi.it (L.M.); cagliardi@dongnocchi.it (C.A.); dcaputo@dongnocchi.it (D.C.); fguerini@dongnocchi.it (F.R.G.); 5 Department of Translational Medicine, CAAD (Center for Translational Research on Autoimmune and Allergic Diseases), University of Eastern Piedmont, 28100 Novara, Italy; francesco.favero@med.uniupo.it (F.F.); davide.cora@uniupo.it (D.C.); 6 Department of Translational Medicine, IRCAD (Interdisciplinary Research Center of Autoimmune Diseases), University of Eastern Piedmont, 28100 Novara, Italy; domizia.vecchio@gmail.com (D.V.); cristoforo.comi@med.uniupo.it (C.C.); 7 Institute of Biomedical Technologies, Bari Unit, National Research Council, 70126 Bari, Italy; maria.liguori@cnr.it; 8 Vita-Salute San Raffaele University, 20132 Milan, Italy; comi.giancarlo@hsr.it (G.C.); filippi.massimo@hsr.it (M.F.); 9 Neurology Unit, IRCCS San Raffaele Scientific Institute, 20132 Milan, Italy; martinelli.vittorio@hsr.it; 10 Neurorehabilitation Unit, IRCCS San Raffaele Scientific Institute, 20132 Milan, Italy; 11 Neurophysiology Service, IRCCS San Raffaele Scientific Institute, 20132 Milan, Italy; 12 Neuroimaging Research Unit, Division of Neuroscience, IRCCS San Raffaele Scientific Institute, 20132 Milan, Italy; 13 Dipartimento di Emergenza e Area Critica, UO Neurologia, Fondazione IRCCS Casa Sollievo della Sofferenza, San Giovanni Rotondo, 71013 Foggia, Italy; m.leone@operapadrepio.it; 14 Department of Pathophysiology and Transplantation (DEPT), Dino Ferrari Centre, Neuroscience Section, University of Milan, 20122 Milan, Italy; filippo.martinelli@unimi.it; 15 Neurology Unit and MS Centre, Foundation IRCCS Ca’ Granda Ospedale Maggiore Policlinico, 20122 Milan, Italy

**Keywords:** multiple sclerosis, multiplex families, linkage study, NGS, rare variants

## Abstract

Known multiple sclerosis (MS) susceptibility variants can only explain half of the disease’s estimated heritability, whereas low-frequency and rare variants may partly account for the missing heritability. Thus, here we sought to determine the occurrence of rare functional variants in a large Italian MS multiplex family with five affected members. For this purpose, we combined linkage analysis and next-generation sequencing (NGS)-based whole exome and whole genome sequencing (WES and WGS, respectively). The genetic burden attributable to known common MS variants was also assessed by weighted genetic risk score (wGRS). We found a significantly higher burden of common variants in the affected family members compared to that observed among sporadic MS patients and healthy controls (HCs). We also identified 34 genes containing at least one low-frequency functional variant shared among all affected family members, showing a significant enrichment in genes involved in specific biological processes—particularly mRNA transport—or neurodegenerative diseases. Altogether, our findings point to a possible pathogenic role of different low-frequency functional MS variants belonging to shared pathways. We propose that these rare variants, together with other known common MS variants, may account for the high number of affected family members within this MS multiplex family.

## 1. Introduction

Multiple sclerosis (MS) is an autoimmune multifactorial disease characterized by the formation of demyelinating lesions in different areas of the central nervous system (CNS) [1]. Both genetic and environmental factors can contribute to the etiology of the disease, with over 200 common non-HLA genetic variants linked to MS susceptibility [2], as well as the well-established association with the HLA-DRB1*1501 allele within the major histocompatibility complex (MHC) region.

Despite the success of genome-wide association studies (GWAS) in pinpointing common single-nucleotide polymorphisms (SNPs) associated with various diseases, known common variants can only account for a small percentage of the estimated heritability of common complex diseases. This has led to the hypothesis that common inherited variations are unlikely to explain the missing heritability of most complex diseases, including MS, pointing to the possible role of different rare and low-frequency (MAF < 5%) variations in contributing to disease susceptibility [3,4]. Indeed, a recent study has shown that up to 5% of MS heritability may be accounted for by low-frequency variants, identifying three novel low-frequency signals driving MS risk independently of common variant signals [5]. Consistent with a polygenic model of MS inheritance, a systematic meta-analysis of published recurrence risk data has shown that the known MS loci account for only up to half of MS heritability [6].

Different strategies have been used to identify MS-associated rare variants, such as the association studies of large case-control cohorts and the analyses of multiplex families (those with at least two MS cases), looking for variants shared among affected individuals within each family. It is in fact known that in MS, similarly to other multifactorial diseases, relatives of affected individuals display an increased disease prevalence, with 15–20% of the patients reporting a family history of the disease [7,8]. However, one major limitation of these approaches is the extremely low number of MS multiplex families comprising several affected individuals—only 0.2% of MS families have reported four or more MS patients [9]. Notwithstanding, several linkage studies on multiplex MS families have been performed using genome-wide polymorphic markers [10,11]. In particular, a study performed by the International Multiple Sclerosis Genetic Consortium [12] showed a significant linkage in the MHC region and two suggestive linkages in chromosomes 17q23 and 5q33 [12].

A more promising approach that has been recently adopted to identify rare MS-associated genetic variants consists of analyzing multiplex MS families through next-generation sequencing (NGS) technology, in particular, whole exome sequencing (WES) and whole genome sequencing (WGS) [13,14,15].

The purpose of the present study was to investigate the role of low-frequency and rare variants, together with known common MS variants in one MS multiplex family with five affected members, representing one of the largest MS multiplex families described in the literature. To this end, we combined traditional techniques, such as weighted genetic risk score (wGRS) and linkage analysis, with NGS-based technologies (i.e., WES and WGS). We report the identification of gene sets containing rare variants shared among these five MS family members enriched in genes involved in specific biological and pathological processes.

## 2. Materials and Methods

### 2.1. Index Family

**Figure 1:**
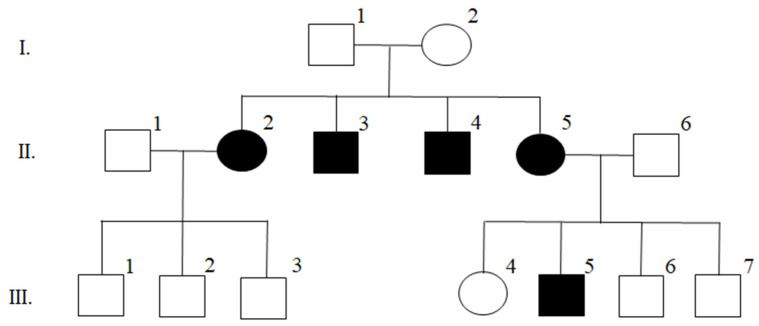
Index family pedigree. Individuals with shaded symbols are affected by MS.

The MS multiplex family enrolled and clinically characterized at the Multiple Sclerosis Unit of IRCCS Santa Maria Nascente—Fondazione Don Gnocchi (Milan) (Figure 1), is composed of five affected individuals on two generations (II.2,3,4,5 and III.5). Clinical details of the family members are reported in Table S1. Briefly, all patients presented at onset with a relapsing-remitting course (RRMS), but II.,2,3,4 evolved to secondary progressive disease. Patient II.2 was also subsequently diagnosed with a comorbidity of systemic lupus erythematosus.

Individual III.2 was diagnosed with sensorineural hearing loss and showed no radiological or immunological signs of MS, whereas individuals I.1,2 and III.1,2,4,6,7 were healthy members of this family.

In the cohort of the non-familial cases analyzed for the genetic risk score, regarding the main phenotypic features, we observed a mean (±standard deviation) age at onset of 33.13 years (±11.02), a relapsing remitting disease onset in 78.7% of the patients, and a mean (±standard deviation) of the MSSS (multiple sclerosis severity score), a standard severity score corrected by the disease duration, of 4.48 (±3.09). The phenotypic features of the affected members of the index family, detailed in Table S1, are in line with these observations, with the notable exception of a higher MSSS value than the mean value of sporadic cases in four out of five family members, possibly indicating a more severe disease in this family.

The study was conducted according to the guidelines of the Declaration of Helsinki, and was approved by the institutional review board of the Don Carlo Gnocchi ONLUS Foundation, Milan (Protocol No. 09_18-06-2014). Informed consent was obtained from all enrolled subjects.

All the wet lab analyses presented in the manuscript have been performed on genomic DNA purified by standard methods, from peripheral whole blood.

### 2.2. Weighted Genetic Risk Score

The weighted genetic risk score (wGRS) of seven family members (I.2; II.1,2,3,4; III.3,5) was calculated as the weighted sum of MS risk alleles by using log odds ratios as weights, as already described by De Jager et al. [16]. The common MS-associated variants, namely 42 non-HLA SNPs and 8 HLA markers—risk alleles DRB1*15:01, 03:01, 13:03, 08:01, and the protective alleles A*02:01, B*44:02, B*38:01; B*55:01—were employed to estimate the cumulative disease risk for each individual given by a representative number of known MS susceptibility loci. The same score was also calculated in an Italian cohort of 595 MS patients with no family history of the disease, and in 820 healthy controls (HCs).

Genotypes for the non-HLA loci for the seven members of the MS index family (I.2; II.1,2,3,4; III.3,5) and the case-control cohort were obtained by exome chip genotyping, as published elsewhere [5]. The 42 SNPs are known MS susceptibility loci (IMSGC, 2013). Each locus is represented by the SNP itself or by a proxy SNP (𝑟^2^ > 0.6). Proxy search was performed with SNAP (https://archive.broadinstitute.org/mpg/snap/ldsearch.php, accessed on 1 June 2017). Of all the susceptibility variants reported by IMSGC [17], only those with a proxy in the exome chip genotyping platform were included in the score. The weight of each variant is the natural log of the OR reported by IMSGC. The list of the 42 non-HLA markers used, with their OR, the proxy used (if any), and the *r^2^* between the proxy and the main SNP is reported in Table S2.

HLA alleles for the case-control cohort were imputed from SNP genotype data using HLA*IMP:2 software [18]. Moreover, complete HLA typing for the index family members (I.1,2; II.2,3,4,5; III.3,4,5) was obtained through the single specific primer-PCR (SSP-PCR) method according to the manufacturer’s instructions (BAG, Lich, Germany, AstraFormedic, Milan, Italy). We used the loci and the odds ratios (OR) reported by Moutsianas [19]. For the protective alleles, we considered any allele different from the protective one as a risk allele, therefore, we correspondingly considered the reciprocal of the reported OR of the protective allele as weight.

The mean wGRS values in the three groups (i.e., family members, non-familial MS, and HCs) were compared using a two-tailed t-Student test for independent samples.

All the wet lab analyses presented in the manuscript have been performed on genomic DNA purified by standard methods, from peripheral whole blood.

### 2.3. Whole Exome Sequencing (WES) and Filtering Pipeline

Six family members (II.,2,3,4,5; III.3, III.5) (Figure 1) were analyzed through WES. Multiplex analysis was carried out using an Ion AmpliSeq™ Library Kit for NGS libraries preparation and an Ion Xpress™ Barcodes Adapters kit according to the manufacturers. The template amplification was performed with the Ion OneTouch System (Thermo Scientific, Waltham, MA, USA). The samples were loaded on an Ion Proton Sequencer (Thermo Scientific, Waltham, MA, USA), Ion Torren Technology.

Coverage details of WES reads of different members of the index family according to the gene position have been reported in Supplementary Figure S1.

The alignment of the reads to the reference genome (Human GRCh37/hg19), the variant calling, and the analysis of the run quality parameters were all performed using the Torrent Suite™ software. Annotation of the variant calling format (VCF) files was performed using Annovar [20] and webAnnovar (https://wannovar.wglab.org/, accessed on 1 January 2021). Prediction of changes in non-canonical splice sites was performed using SpliceAI tool [21]. We also checked clinical significance of the variants through the Varsome database (https://varsome.com/, accessed on 1 August 2021).

All variants shared by all the affected members of the index family were filtered according to frequency and putative functional impact. Regarding frequency, we selected variants with MAF < 5% or absent in the following databases: 1000 Genomes (European population); GnomAD (non-Finnic European population); and/or ExAC (non-Finnic European population). To account for functional impact of the variant, we applied different filtering criteria based on the variant type.

- For missense variants, we prioritized those predicted as damaging by the following predictors: CADD_Phred > 10 [22], or those rated by two out of the following five tools specific for missense variations: SIFT [23]; POLYPHEN [24]; Mutation Taster [25]; Mutation Assessor [26]; FATHMM [27].
- For synonymous variants, we selected variants presenting with at least one of these predictors: CADD > 10 [22]; GWAVA > 0.5 [28]; GERP > 4 [29]; SPIDEX < –4 or > +4 [30]; SPLICE AI > 0.4 [21].
- All shared low-frequency variants belonging to categories with an expected high functional impact (i.e., splicing, nonframe/frameshift indels, stopgain, stoploss variants) were selected.
- For non-coding (i.e., ncRNA, UTR, intronic, upstream) variants, we selected those with at least one of these predictors: CADD > 10 [22]; GWAVA > 0.5 [28]; GERP > 4 [29]; SPIDEX <−4 or > +4 [30]; SPLICE AI > 0.4 [21].

### 2.4. Linkage-Analysis

We performed a non-parametric whole-genome linkage analysis on I.2; II.2,3,4,5; III.3,5 individuals of the index family using:

(a) 42,764 variants whose genotypes were inferred from the WES data (for II.1,2,3,4; III.3,5 individuals); and
(b) 23,443 variants previously obtained from a genotyping platform (exome chip, IMSGC, Cell, 2018) containing 247,871 SNPs, mainly derived from published exome sequencing studies also including rare variants. Standard quality controls were applied as previously described [5] (for I.2; II.1,2,3,4; III.3,5 individuals).

For variants that were inferred from the WES data, the VCF file was converted into genotype calls using VCFtools. To increase the precision of this phase, we included four samples in the analysis unrelated to the index family that had been sequenced in the same sequencing experiment, and merged the VCF files of the various patients in a single multi-sample VCF file. After removing overlapping variants, the genotype file obtained from WES data was merged with data obtained from the exome chip genotyping platform, using PLINK software [31]. We obtained a merged dataset of 57,241 variants.

For each variant, we instructed the software with both genetic map and allele frequency data. Build *GRCh37 (Genome* Reference Consortium Human *Build* 37) genetic map data were obtained from the HapMap Phase II genetic map [32]. Allele frequency data for the WES variants were obtained from public databases—when available, we used GnomAD non-Finnic Europeans frequency, otherwise, we used ExAC non-Finnish Europeans or 1000 genomes-Europeans frequencies. For the genotyping platform variants, we employed the allele frequencies observed in an Italian cohort of 1749 samples (925 MS patients and 824 HCs).

Non-parametric linkage analysis was performed using Merlin v.1.1.2 software [33]. We calculated Whittenmore and Harpen NPL all statistics. As we were performing linkage analysis on only one multiplex family, we choose the Kong and Cox exponential model instead of the Kong and Cox linear model. The maximum possible scores using our dataset correspond to a Kong and Cox exponential LOD score (exLOD) = 1.806, and a Kong and Cox *p*-value (K&C p) = 0.00196.

To identify linkage regions, we regarded any variant reaching the maximum exLOD = 1.806 as linkage peaks. For each peak, we identified a shore, defining shore boundaries as the variant for which exLOD drops to 0.

### 2.5. Whole Genome Sequencing (WGS) and Filtering Pipeline

In addition to WES, a WGS analysis was performed for one of the affected members (individual II.3) of the index family. The sequencing reaction and standard bioinformatic pipeline was applied by the Theragen sequencing facility. This facility prepared the whole genome library according to a “PCR-free” condition, and performed paired-end sequencing using an Illumina HiSeqX, producing 2 × 150 bp read lengths at 30x coverage. The reads were aligned on the human GRCh37/hg19 reference genome using BWA software [34]. The variant calling was performed with GATK software (https://gatk.broadinstitute.org/hc/en-us, 2019).

We searched for WGS low-frequency (MAF < 5% or absent in public databases) functional variations located in the linkage regions, using filtering criteria similar to those applied for the WES analysis:

- for all the coding variants, we applied the same filtering pipeline described for WES.
- for non-coding (ncRNA, UTR, intronic, upstream) variants, we selected those with a SpliceAI score > 0.4 [21]. SpliceAI is a deep learning-based tool that allows the identification of putative splice variants.

In addition to the single nucleotide variants (SNV) and indels called by the above tools, we utilized CNVkit to call structural variants [35]. CNVkit uses both the targeted reads and the nonspecifically captured off-target reads to infer copy number evenly across the genome.

### 2.6. Association of Single Variants in Large International Datasets

To check for single-variant association with disease susceptibility, we took advantage of the summary statistics from genomic association studies performed by three large international datasets typed with genotyping platforms: MS Immunochip [17]; MS Exome Chip [5]; MS Chip [2].

### 2.7. Gene-Based Burden Analysis

The genes harboring the variants selected with the above pipelines were selected for further investigations. On these genes, we performed gene-based burden analysis on a cohort of 28 multiplex MS Italian families—not including the index family—with at least 3 affected members and no shared geographic origin, and 30 unrelated HCs (multiplex sample set), which were investigated by WES. The whole cohort was composed of 150 subjects: 81 MS patients and 69 unaffected subjects. Libraries were prepared using exome enriched kits: a TruSeq Exome Kit (Illumina^®^, San Diego, CA USA) for two families; a SureSelect QXT Human All Exon V5 kit (Agilent, Santa Clara, CA USA) for 25 families; and a TruSeqTM DNA Sample Preparation Kit (Illumina^®^) for one family. Libraries were sequenced on the HiSeq 2500 platform (Illumina^®^).

The gene-based burden analysis was performed filtering variants with criteria similar to those applied on our index family, based on allele frequency and predicted functional impact, as described [36].

The aggregate contribution of variants within prioritized genes was evaluated through gene-based burden analysis using the PedGene tool [37], which exploits pedigree-based kinship matrices to account for shared genetic ancestry among family members.

### 2.8. Enrichment Analysis

We performed enrichment analysis of the selected genes using two different tools based on functional annotations and protein interaction networks: Metascape Analysis Report Package (https://metascape.org/gp/index.html#/main/step1, accessed on 1 July 2021) and ToppGene Suite (https://toppgene.cchmc.org/contact.jsp, accessed on 1 July 2021).

## 3. Results

### 3.1. Description of the Index Family and Workflow of the Study

The Italian MS multiplex family enrolled in this study (index family, Figure 1) is composed of five affected individuals on two generations (II.2,3,4,5 and III.5). At disease onset, all patients presented with a relapsing-remitting course (RRMS), with three of them (II.,2,3,4) evolving to secondary progressive disease. Both parents of the first generation (I.1 and I.2), as well as the other healthy members of this family, showed no MS disease sign.

**Figure 2:**
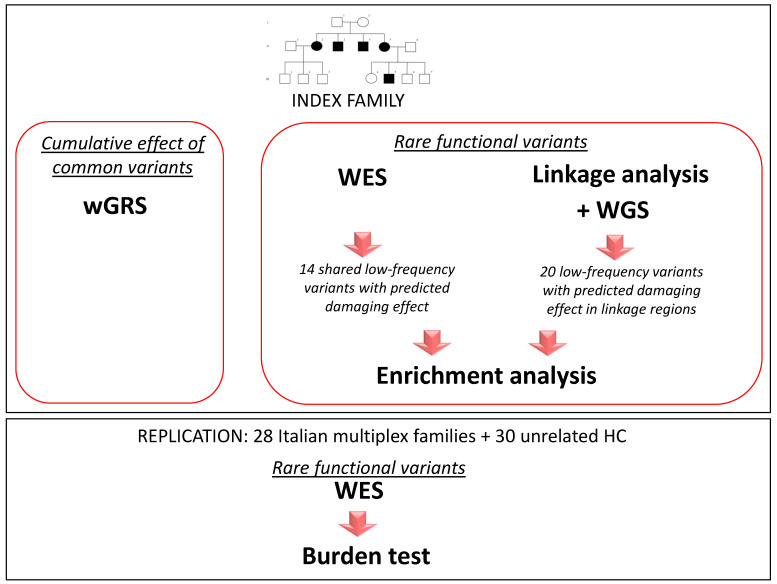
Study workflow.

To comprehensively explore the genetic architecture possibly accounting for the high number of affected family members within this pedigree, we investigated the role of both common and rare variants following the workflow of the study presented in Figure 2. Briefly, we assessed the genetic risk conferred by known common MS variants in the family by wGRS, while we combined linkage analysis with NGS-based technologies, performing WES on all the affected individuals of the family, and WGS analysis on one affected individual, to explore the possible role of low-frequency and rare variants with a predicted functional role according to in silico tools. We next evaluated whether the genes carrying the functional low-frequency variants were enriched in genes involved in certain molecular pathways. Finally, as an initial attempt to reproduce our findings, we assessed whether the genes carrying the functional low-frequency and rare variants shared by all affected family members harbored variants with similar features to those present in an independent dataset of 28 Italian multiplex families.

### 3.2. Role of Classical HLA Alleles

The family members tested positive for both protective and risk alleles with no clear segregation with disease status (Table S3). In particular, we observed a protective allele (A*02:01) shared among four individuals (one healthy and three with MS) and five risk alleles: DR8 (DRB1*08:01); DR17 (DRB1*03:01); DR13 (DRB1*13:03); DR15 (DRB1*15:01). Each family member was positive for at least one of these risk alleles, regardless of disease status.

### 3.3. Burden of Common MS Susceptibility Variants

**Figure 3:**
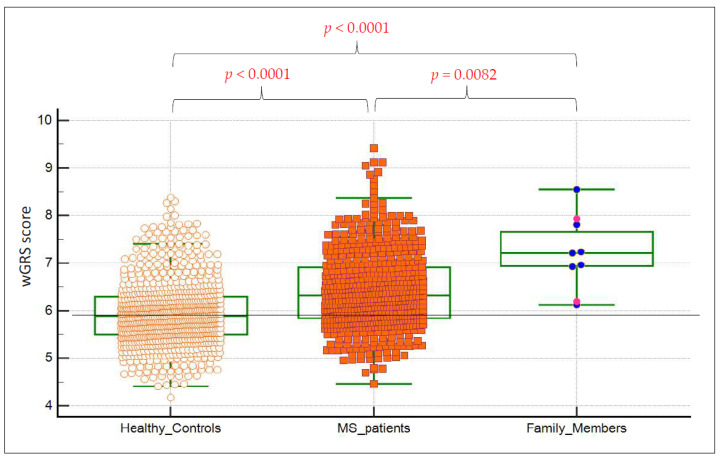
wGRS comparison between index family members, non-familial MS cases, and healthy controls. wGRS analysis of known common MS risk alleles. wGRS was constructed with results of 42 non-HLA SNPs and 8 HLA markers (risk alleles DRB1*15:01, 03:01, 13:03, 08:01, and the protective alleles A*02:01, B*44:02, B*38:01; B*55:01). Within the family member category, two healthy members (I.2 and III.3) were included in the distribution (bright pink dots), but they were excluded from the statistical analysis.

To ascertain the role of known common MS-associated variants, we tested the combined disease risk by constructing a wGRS with 42 non-HLA SNPs and 8 HLA loci in the index family, as well as in 819 (HCs) and 598 MS patients with no MS family history (Figure 3). The affected family members had a significantly higher genetic risk score in comparison with 820 (HCs) (*p* < 0.0001) and 598 MS non-familial cases (*p* = 0.0082). Interestingly, both tested healthy family members showed a higher wGRS than the mean value observed in the control population (Figure 3). Altogether, these results suggest a role of known common MS-associated variants in augmented MS risk.

### 3.4. Identification of Shared Low-Frequency Functional Variants in the Index Family by Whole Exome Sequencing

To test the role of rare and low-frequency functional variants, we performed WES on all five affected family members, and we filtered all the variants shared among all the five affected members according to frequency and in silico functional prediction (see Material and Methods). We filtered 70 low-frequency (MAF < 0.05) variants shared by all affected members with a putative functional effect located in 71 different genes. Based on the association with MS susceptibility, these variants were subsequently prioritized in three large international case/control MS datasets: (1) Exome Chip cohort, IMSGC, Cell, 2018; (2) MS Chip discovery cohort, IMSGC, Nature, 2019; (3) Immunochip cohort, IMSGC, Nat Genet, 2013. More specifically, we filtered out variants whose association with MS susceptibility was not significant among any of these cohorts, whereas we only retained variants that were at least nominally significant or absent in any of the aforementioned large MS datasets. We selected 14 variants in 14 genes: 7 missense, 1 splicing, 4 synonymous, and 2 non-coding variants (see Table S4).

### 3.5. Linkage Analysis on Index Family

To identify genomic regions shared among the affected members of the family, we next performed non-parametric whole-genome linkage analysis. For this purpose, we used two sample sets: (a) 42,764 variants whose genotypes were inferred from the WES data; and (b) 23,443 variants previously obtained from a genotyping platform [5]. After removing overlapping variants, we obtained a merged dataset of 57,241 variants. Eighty-four genomic regions were shared among all affected members, showing the highest linkage values (exponential LOD = 1.806), the highest obtainable with our sample size. The boundaries of the regions were defined considering the marker where the exponential LOD dropped to 0. These 84 regions accounted for a total of about 186.5 Mb.

### 3.6. Identification of Low-Frequency Functional Variants in Linkage Regions

WGS analysis was performed on one affected member of the index family (II.4, Figure 1) using a similar filtering pipeline employed for the WES data based on frequency and in silico functional prediction (see Materials and Methods). In the 84 linkage regions, we filtered 52 low-frequency variants with a predicted functional role, located in 50 genes. Among these, 32 variants were removed because they are not significantly associated with MS susceptibility in the three following international cohorts: (1) Exome Chip, [5]; (2) MS Chip discovery [2]; and (3) Immunochip [17]. Accordingly, we selected 20 variants in 20 genes: 3 frameshift; 3 non-frameshift deletions; 1 splicing; 7 missense; 1 synonymous; and 5 non-coding variants (Table S5).

### 3.7. Gene-Based Analysis of Identified Genes in an Independent Cohort

Our dual strategy based on WES and linkage-WGS led to the selection of 34 genes for further analysis (Tables S4 and S5). These genes were subjected to gene-based burden analysis using a sample set of 28 multiplex MS Italian families and 30 unrelated HCs (multiplex sample set), totaling 81 MS patients and 69 unaffected subjects analyzed by WES. The 34 selected genes were filtered on this sample set using similar criteria to those applied to our index family, which were based on allele frequency and predicted functional impact. This led to the selection of 65 low-frequency variants with a putative functional role (Table S6).

Of the 34 selected genes, 16 genes tested positive for at least one low-frequency functional variant in at least one of the MS families, while 14 genes tested positive for at least two of such variants. For these 14 genes, we tested the aggregate contribution of low-frequency damaging variants via gene-based burden test (Table 1), without detecting any statistically significant burden. Thus, among the 16 genes analyzed, we identified a total of 65 variants meeting our selection criteria.

Of note, the burden test was not performed for the two genes *CCNL2* and *TMX3*, as they only displayed one variant satisfying the filtering criteria (Table 2). For *CCNL2*, the variant shared by the affected members of the index family, a five base deletion involving the splice acceptor sit was also observed in the multiplex sample set, specifically in twelve MS patients belonging to six different multiplex families, five healthy relatives of the patients, and five unrelated healthy subjects. This variant, which is reported in public databases with a frequency between 0.040–0.049 in non-Finnish Europeans, was the only one detected in both the index family and the multiplex sample set. This variant is classified as benign by Varsome software, according to the standard clinical criteria proposed by the American College of Medical Genetics (ACMG) [38].

*TMX3* was selected because of a shared intronic variant with high CADD and SPIDEX scores. In the multiplex sample set, we observed a substitution in the splice donor site of this gene in two patients from two distinct MS multiplex families. This variant is classified as pathogenic by Varsome software, according to the standard ACMG criteria [38]. Of note, the *TMX3* gene appears as the only gene prioritized by both additional analyses (Supplementary Material S1). More specifically, it was one of the three genes carrying a variant not shared by the healthy family member of the index family (III.3) among the total 14 genes prioritized in Table S4 and shared by all affected family members. Furthermore, it is the only gene carrying a common functional variant in compound heterozygosity in a patient also positive for a rare functional variant.

### 3.8. Enrichment Analysis of Genes Carrying Selected Low-Frequency Functional Variants Shared within the Index Multiplex Family

We next asked whether, among the list of 34 variant-harboring genes, there would be a peculiar enrichment in shared biological and/or pathological processes. To address this important issue, we performed enrichment analysis using two different tools: Metascape Analysis Report Package (https://metascape.org/gp/index.html#/main/step1, accessed on 1 July 2021) and ToppGene (https://toppgene.cchmc.org/contact.jsp, accessed on 1 July 2021) (Table 3). Both tools detected a statistically significant enrichment in genes involved in mRNA transport (5 genes out of 34). Of note, three of these five genes (i.e., *SMG1*, *HSF1*, *MYO1C*) are regulated by the same transcription factor (RFX1_01). Furthermore, ToppGene analysis detected a significant enrichment in genes regulating motor activity and transcription coactivator activity. Finally, our analysis revealed another enrichment in genes linked to susceptibility/etiology of neurodegenerative disorders and diseases of the nervous system.

## 4. Discussion

The aim of this study was to carry out a comprehensive analysis of the genetic architecture possibly accounting for the high number of affected family members within a large MS multiplex family comprising five affected members, mainly focusing on rare and low-frequency variants. Our ultimate goal was to identify low-frequency genetic variants associated with increased MS susceptibility in the general population.

Despite the success of GWAS in finding common SNPs associated with disease, the MS-associated loci identified so far account for only half of the genetic component involved in MS. Thus, we still lack a complete understanding of the mechanisms at the basis of MS susceptibility. In particular, the role of rare variants associated with MS remains largely unexplored mainly due to technical challenges. It is in fact quite difficult to identify these rare signals because they require a high statistical power that can only be achieved with large cohorts. In addition, the analysis of these variants generally requires the implementation of NGS-based techniques, which are more complex and expensive than those utilized to genotype common variants.

In this scenario, the study of multiplex families constitutes an alternative suitable strategy to identify rare variants associated with complex diseases. A classical approach for the genetic characterization of MS multiplex family is linkage analysis, which has led to several studies [10,11,12,13]. Unfortunately, linkage analyses in MS have not yet identified unequivocal linkage regions, except for the HLA region. Nevertheless, these studies have succeeded in identifying suggestive regions of potential linkage that will obviously require more in-depth investigations to pinpoint the specific genes associated with the disease. For example, a large linkage study conducted on 730 multiplex families [12] identified a suggestive linkage in the chromosome region 17q23, which overlaps with the region containing an association signal identified in a later association study [17]. This proves that linkage analysis can be a powerful tool to detect disease-causing variants also for complex diseases like MS, especially when coupled with NGS data and performed on large multiplex families.

In its traditional formulation, linkage analysis typically uses a few hundred microsatellite markers, but it can also be successfully performed using SNPs. In MS, for instance, the IMSGC performed a linkage study using 4,506 SNP markers [12]. In the present study, we employed both SNP genotype data from genotyping platforms and variants from WES to construct a large set of variants that were used for linkage analysis. The ensuing results were then coupled with WES and WGS data to identify low-frequency functional variants shared by the affected members of the family analyzed. Likewise, Gazal et al. [39] demonstrated that WES-extracted genotypes could be reliably used for linkage analysis, showing a similar performance compared with genotyping platform data. Furthermore, several studies have recently employed WES- or WGS-derived data to perform linkage analysis, also on complex diseases [40].

The combination of NGS data with linkage analysis to perform deep sequencing of linkage regions has already been employed for several mendelian diseases, leading to an increase in the identification of genes involved in their pathogenesis [41,42]. More recently, the same strategy has also been applied to complex diseases, including MS. For example, in a recent study [13], a large MS Dutch family with 11 affected individuals was analyzed by coupling linkage analysis with WES, leading to the identification of a rare missense variant located in the FKBP prolyl isomerase family member 6 (*FKBP6*) gene, segregating with the disease. However, the same variant was not associated with disease risk in a case/control cohort. In another study published in 2017 [14], a rare three-variant haplotype in the P2X purinoceptor 7 (*P2RX7*) and P2X purinoceptor 4 (*P2RX4*) genes was identified, which segregated with MS within the family analyzed.

In the present study, we initially evaluated the contribution of known common genetic variants to the increased MS risk observed in this family. To this end, we performed wGRS on 8 HLA loci and 42 non-HLA loci, comparing wGRS values of the affected members of the family with those observed in the general healthy Italian population and in Italian non-familial MS cases. These analyses show that the index family members have a statistically significantly higher genetic risk score compared to HCs, confirming the general finding that MS patients have a much higher MS risk score than that of HCs. More importantly, these results indicate that disease susceptibility in this family is at least partially driven by a genetic background given by known common MS risk variants, including HLA and non-HLA variants.

In the second part of our study, we sought to determine the added role of rare functional variation to the genetic burden in this MS multiplex family. To search for low-frequency variants that may contribute to the high genetic burden of this family, we followed a two-pronged strategy: (i) searching for rare functional variations shared among the affected family members by WES; and (ii) identifying linkage regions that have been more deeply scanned through the WGS performed on one family member. Indeed, the combined acquisition of WGS allowed us a better evaluation of non-coding regulative regions, as well as an evaluation of complex genomic structural variants such as copy number variants (CNV).

WES- and WGS-derived variants were selected based on the following criteria: segregation in all affected family members; frequency; predicted function; and association with MS susceptibility previously observed in three large international case/control cohorts. The MS susceptibility variants that have thus far emerged from GWASs are mainly located in the regulative regions. A recent large-scale genomic study, well-powered to detect associations with rare variants, detected seven low-frequency MS susceptibility variants [5]. Among these, only one variant showed a frequency below 1%, while five had frequencies ≥3.9%. For these reasons, we decided to adopt a “conservative strategy” for the filtering of NGS variants, not only focusing on rare variants with strong functional effects, but also including variants with frequencies as high as 5%, synonymous variants, and variants located in regulative regions.

To identify variants with a putative functional role, we took advantage of an in silico prediction tool battery that allowed us also to estimate the possible effect of intronic variants, SpliceAI, which calculates the predicted impact of a variation on splicing using a deep learning-based approach. This choice has led to the selection of some variants that do not have a strong pathogenic effect, but that can still play some putative role in a multifactorial disease such as MS. Even though most of the selected variants are classified as “uncertain function” or “likely benign” according to the standard clinical classification proposed by the ACMG [37], we observed a frameshift insertion in the heat shock transcription factor 1 (*HSF1*) gene—located in a linkage region—classified as likely pathogenic. Interestingly, for this gene, we observed a different frameshift deletion classified as pathogenic in one of the multiplex families of the replication cohort. *HSF1* codes for a transcription factor that is rapidly induced after temperature stress and can bind to heat shock promoter elements. HSF1 is strongly involved in tumorigenesis and tumor progression, and it can drive migration, invasion, and anchorage-independent growth of cancer cells [43]. Relevant to MS predisposition, HSF1-induced heat shock proteins play an immunomodulatory role by acting as both pro-inflammatory and anti-inflammatory triggers [44]. Intriguingly, Dello Russo et al. have shown that pharmacological induction of the heat shock response in experimental autoimmune encephalomyelitis (EAE) mice (the canonical MS mouse model) prevents disease onset and reduces clinical symptoms [45]. Furthermore, it has been reported that, after heat shock, the heat shock protein Hsp70 is upregulated six-fold higher in MS patients compared to HCs [46], suggesting a possible link between aberrant Hsp70 activity and autoimmunity in MS.

With regard to the 34 genes containing at least one shared, low-frequency functional variant, we set up a replication in an independent cohort of 28 multiplex MS Italian families and 30 unrelated HCs through a gene-based burden test. None of the 34 genes showed a significant unbalance of rare variants in MS patients vs. unaffected individuals.

One of the limitations of this study is that besides the demonstration of the statistically significant higher burden of well-established MS common variants, we were unable to formally demonstrate the role of low-frequency and rare variants. Indeed, we did not identify a clear pathogenic variant shared by all affected family members, since the family structure does not allow a statistically significant linkage to be reached. In addition, we failed to observe a statistically significant replication in the multiplex families of the replication phase. Nevertheless, among those genes shared among all five affected family members of the index family containing variants with some predicted functional impact, we report a statistically significant enrichment in genes involved in the same biological processes—especially mRNA transport—and interestingly, a significant enrichment in genes implicated in neurodegenerative diseases. It is thus likely that some of these variants may contribute to the burden given by common variation, but without a strong effect.

The hypothesis that common and rare genetic variants both contribute to the familial aggregation of large multiplex families has been demonstrated for other complex diseases, with several examples derived from psychiatric diseases [47].

## 5. Conclusions

In conclusion, for the large MS multiplex family analyzed in the present study, we hypothesize a possible pathogenic role of low-frequency and rare variants, in addition to the susceptibility provided by common well-established MS variants.

Further studies are needed to replicate these findings and to explore whether the high prevalence of MS in this family may be partly due to other shared factors, such as environmental exposure and epigenetic stimulation.

## Figures and Tables

**Table 1:** Gene based burden test analysis of the multiplex cohort.

Gene	Chr	No. of Variants ^a^	Burden ^b^	*p*
*MYO5C*	15	4	−1.6295	0.103214
*NOTCH1*	9	4	−1.3859	0.165765
*PPRC1*	10	5	−1.3563	0.174993
*ABCA1*	9	2	1.2021	0.229316
*DNAH8*	6	7	−1.0906	0.275457
*LOXHD1*	18	10	−0.781	0.434797
*HSF1*	8	3	−0.6585	0.510208
*ADAMTS5*	21	6	0.4399	0.659993
*MYO1C*	17	5	0.3498	0.726526
*ASXL3*	18	4	−0.1488	0.88168
*KIF1A*	2	2	0.1461	0.883868
*TLN2*	15	2	0.1461	0.883868
*ATN1*	12	3	−0.1299	0.896626
*KIF26B*	1	4	0.0279	0.977741

^a^ Total number of low-frequency functional variants observed in the multiplex sample set. ^b^ A positive stat means that the number of rare functional variants is increased in MS patients compared to healthy individuals.

**Table 2:** Genes presenting with only one low-frequency functional variant in a multiplex sample set. For these genes, the burden test was not performed, as they only displayed one rare variant with a predicted functional effect.

Gene	Chr	rs ID	POS (hg19)	REF	ALT	n MS ^a^	n CT ^a^	Effect	Ann Impact ^b^	AF EUR ^c^	CADD Phred ^d^
*CCNL2* ^e^	1	rs3831366; rs368050244	1334052	CTAGAG	C	6 ^f^	5 ^f^	Splice acceptor variant	HIGH	0.0487	NA
*TMX3*	18	chr18:66358530	66358530	A	G	2	0	Splice donor variant	HIGH	−1	24.5

^a^ For every family, only one subject was considered, independently of their health status. ^b^ Predicted impact on protein function. ^c^ Allelic frequency on Europeans. ^e^ The CCNL2 was observed also in the index family. ^f^ The deletion was observed in 12 MS patients belonging to 6 different multiplex families, 5 healthy relatives of the patients, and 5 unrelated healthy subjects. ^d^ Phred CADD score.

**Table 3:** Enrichment analysis. The table shows the results obtained using ToppGene software.

					ToppGene		
Category	GO Term	Description	Genes	No. of Selected Genes	No. of Hits in Genome	*p*	*p* Adjusted
Biological processes ^a^	GO:0051028	mRNA transport	*AGFG1,HSF1,MYO1C,SMG1,MAGOHB*	5	157	6.2 × 10^−6^	0.011
Molecular function	GO:0003774	motor activity	*MYO1C,KIF1A,DNAH8,KIF26B,MYO5C*	5	134	3.44 × 10^−6^	9.50 × 10^−4^
Molecular function	GO:0003713	transcription coactivator activity ^b^	*MMS19,ATN1,PPRC1,NOTCH1,RNF20*	5	285	1.29 × 10^−4^	3.57 × 10^−2^
Disease	C0524851	Neurodegenerative Disorders	*AGFG1,PPP2R2B,ABCA1,GRIN2B,ATN1,TDP1,HSF1,KIF1A,DNAH8,LDLR,NOTCH1*	11	1480	1.01 × 10^−5^	0.0156
Disease	C0027765	nervous system disorder ^c^	*AGFG1,SMG1,PPP2R2B,ABCA1,GRIN2B,TDP1,KIF1A,NOTCH1,RNF20*	9	942	1.12 × 10^−5^	0.0173
Transcription factors		RFX1_01 ^d^	*SMG1,PPP2R2B,HSF1,MYO1C*	4	194	7.82 × 10^−5^	0.0375

^a^ The same five genes also resulted significantly enriched in the mRNA transport pathway according to Metascape software: No. of hits in genome = 152; *p* = 1.05 × 10^−6^; *p* adjusted = 0.024. ^b^ Two genes (MMS19 and PPRC1) are located in the same linkage region. Enrichment analysis was re-run excluding one of the genes. The term still shows a nominal enrichment (*p* = 0.00105, but it is no longer significant after applying Bonferroni correction. ^c^ Two genes (ABCA1 and RNF20) are located in the same linkage region. Enrichment analysis was re-run excluding one of the genes. The term still shows a nominal enrichment (*p* = 3.03 × 10^−4^), but it is no longer significant after applying Bonferroni correction. ^d^ Two genes (PPP2R2B and YIPF5) are located in the same linkage region. Enrichment analysis was re-run excluding one of the genes, but it is not significant. No. of hits in genome: number of genes in genome linked to the specific GO Term. For *p* adjusted, Bonferroni correction was performed, multiplying for the number of annotations in the category.

## Supplementary Materials

The following are available online at https://www.mdpi.com/article/10.3390/genes12101607/s1, Material S1: Supplementary Result. Figure S1: Coverage of WES reads of different members of the index family according to the gene position. Table S1: Clinical features of the members of the index family. Table S2: Non-HLA markers used for wGRS calculation. Table S3: HLA typing and wGRS_HLA in all the members of index family. Table S4: Functional low-frequency variants shared by all affected members of the index family. Table S5: Low-frequency functional variants in LD regions. Table S6: Low-frequency functional variants observed in the replication (multiplex sample set).
