# Supplementary material for: An Investigation of the Role of Common and Rare Variants in a Large Italian Multiplex Family of Multiple Sclerosis Patients
Source: Genes (Basel). 2021 Oct 13;12(10):1607. doi: 10.3390/genes12101607 (PMC8535321; doi:10.3390/genes12101607)

SUPPLEMENTARY MATERIALS

SUPPLEMENTARY RESULTS

Segregation analysis of the selected variants in the index family in a healthy family member. WES was performed in all the affected members of the index family, and on one healthy member (individual III.3).

As multiple sclerosis is a multifactorial disease, we expect to observe variants that may contribute to disease etiology also in the healthy relatives and in the general population, as a consequence of the multigenic component or of the phenomenon of incomplete penetrance. Therefore, to avoid missing any disease related variant, in our study design we chose to select rare variants shared by all the affected members, independently of the status of this variant in the healthy family members.

In addition, to pinpoint variants that are more likely to be involved in disease pathogenesis, we performed a segregation analysis of the 14 variants prioritized in the index family and shared by all affected family members (table S4) in the healthy individual (III.3)

Of the 14 variants from table S4, 11 were shared also by the healthy individual III.3. The remaining three variants were shared only by the affected members: a synonymous variant with a high CADD score in *TLN2* gene, a missense variation in *MYO1C* gene and a IVS16+4 substitution in *TMX3* gene with high CADD and SPIDEX scores.

Identification of common variants with putative functional role in genes carrying prioritized low frequency variants. We checked both the index family and the replication cohort (multiplex sample set) for common variants (MAF > 0.5) on the 16 selected genes that tested positive for at least one low-frequency, functional variant in at least one of the MS families, for identification of compound heterozygous variants with high MAF in other allele in lieu of high prevalence of the disease. For common variants, we applied the same functional filters applied to the rare variants. The identification of genes carrying compound heterozygous variants with high MAF in individual carrying a prioritized low frequency functional variant could support the role of those genes in the disease etiology.

We observed 9 common functional variations: 5 variants in the index family (shared by the affected members), and 4 in the multiplex cohort.

With the only exception of *TMX3* frameshift variation, that was not covered by the genotyping platforms used for the international GWASs, all these variants were tested for association with susceptibility to MS in the same 3 international datasets used to filter rare variants in the discovery phase, and none of them show significant association with MS ($p>0.05$). Therefore, we believe that these are common polymorphisms, not related with MS pathogenesis.

The frameshift variant in the *TMX3* gene shows a 9.0% frequency in Europeans (https://www.ncbi.nlm.nih.gov). The alternative allele of this variant was observed in 15 MS patients (from 10 different families), in 5 of their healthy relatives and in 3 unrelated controls from the multiplex family. One of the patients showing this variant shows also a rare functional variant in compound heterozygosity in the same gene, a substitution in the donor splice site with a predicted pathogenic effect (Varsome).

Table S1 Clinical features of the members of the index family

ID MEMBER	GENDER	AGE	DISEASE ONSET	AGE AT ONSET	SYMPTOMS AT ONSET	DISEASE COURSE	CLINICAL ASSESMENT	EDSS	MSSS	MRI	THERAPY	OLIGOCLONAL BAND (IEF)	OTHER DISEASES
**I.1**	M	deceased				healthy							collagenopathy
**I.2**	F	91				healthy							
**II.1**	M	66				healthy							
**II.2**	F	63	1992	35	sensory	CPII	2016	6.0	5.03	Positive Brain + Marrow +	Azathipirine	Positive	Systemic Lupus Erythemathosus
**II.3**	M	69	1980	28	motor	CPII	2008	7.5	7.90	Positive Brain +	-	Positive	
**II.4**	M	66	1975	20	motor	CPII	2008	7.5	7.54	Positive Marrow +	-	Positive	
**II.5**	F	57	1994	31	cerebellar	RRMS	2016	3.5	2.20	Positive Brain+	-	Positive	Thyroid cancer Bechet syndrome
**II.6**	M	59				healthy							
**III1**	M	39				healthy							
**III.2**	M	37				healthy							sensorineural hearing loss
**III.3**	M	41				healthy							
**III.4**	F	37				healthy							
**III.5**	M	35	2012	26	Motor sensory	RRMS	2014	2.0	5.24	Positive Brain+ Marrow+	Natalizumab	Positive	
**III.6**	M	28				healthy							
**III.7**	M	23				healthy							

RRMS: relapsing remitting multiple sclerosis

CPII: secondary progressive disease

EDSS: Expanded Disability Severity Score

MSSS: Multiple Sclerosis Severity Score

Supplementary Figure S1

Coverage of WES reads of different members of the index family according to the gene position

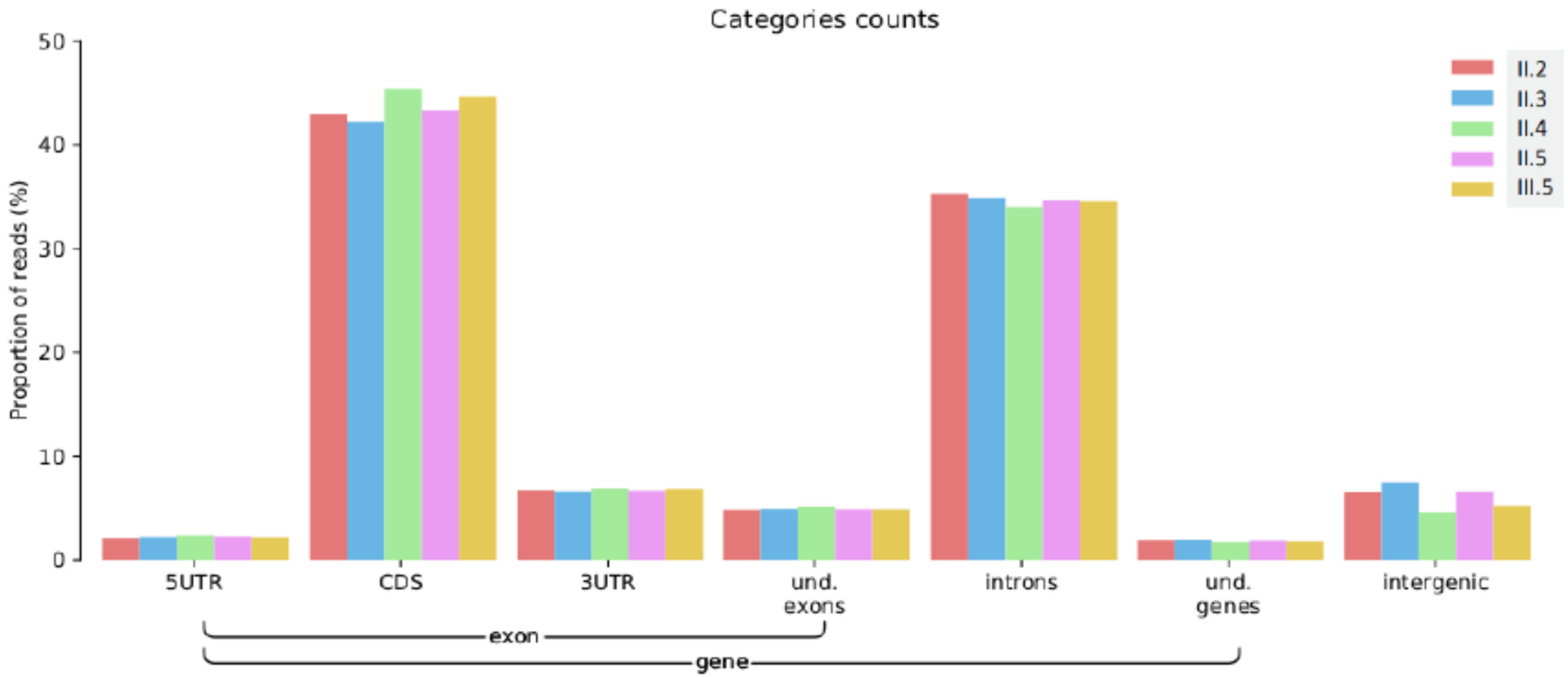

Supplement: Supplementary file 1 [file genes-12-01607-s001.zip › genes-1361747 SI.pdf]
